# A machine learning algorithm to explore the impact of agricultural land-use types on CO_2_ emissions in Vietnam in the period 1990–2019

**DOI:** 10.1371/journal.pone.0350920

**Published:** 2026-06-11

**Authors:** An Thinh Nguyen, Tuong Anh Nguyen, The Hoang Tran, Xuan Truc Doan, Ngoc Trinh Phuong, Tuyen Tran Thi, Ngoc Anh Le, Thao Do Thi, Trang Le Huyen

**Affiliations:** 1 VNU University of Economics and Business, Vietnam National University, Hanoi, Vietnam; 2 Tan Trao University, Tuyen Quang, Vietnam; 3 Sai Gon University, Ho Chi Minh City, Vietnam; 4 RMIT University, Ho Chi Minh City, Vietnam; 5 School of Hospitality and Tourism, Hue University, Hue City, Vietnam; The University of the South Pacific, FIJI

## Abstract

The paper deals with an application of machine learning algorithms to examine the impact of agricultural land-use types on CO₂ emissions in Vietnam during the period 1990–2019. A four-layer Artificial Neural Network (ANN) model was employed to analyze the relationship between 21 agricultural land-use types (LUTs) and CO₂ emissions. The selected LUTs cover major agricultural products, including crops, meat, and vegetables. The results indicate that most agricultural LUTs are positively associated with CO₂ emissions, including bananas, dry beans, cabbages, cashew nuts in shell, fresh cassava, raw or retted jute, cauliflowers and broccoli, dry chilies and peppers, raw cinnamon and cinnamon-tree flowers, coconuts in shell, green coffee, groundnuts excluding shelled, fresh hen eggs in shell, watermelons, tea leaves, and sweet potatoes. By contrast, fresh or chilled horse meat, unmanufactured tobacco, soya beans, sesame seed, and rice show negative associations with CO₂ emissions. These findings provide empirical evidence to support policymakers in designing targeted strategies for reducing greenhouse gas (GHG) emissions from agricultural production in Vietnam.

## Introduction

Agricultural production has long been a vital pillar of Vietnam’s economy, contributing significantly to the country’s gross domestic product (GDP) and supporting the livelihoods of millions. However, this sector also poses serious environmental challenges, particularly in greenhouse gas (GHG) emissions. The expansion and intensification of agricultural activities have increased the demand for land use, leading to higher CO_2_ emissions. Between 1990 and 2019, Vietnam’s CO_2_ emissions experienced a sharp rise, escalating from 62.9 million tons in 1990 to 263.6 million tons in 2019, marking a total increase of approximately 319.1% (World Bank, 2019). While trade liberalization and rapid economic development have driven GDP growth, insufficient attention has been given to the environmental consequences, especially in the agricultural sector.

Agriculture’s role in environmental degradation is well-documented in the literature. Empirical studies have shown that farming practices such as tillage, the use of synthetic fertilizers, and livestock production contribute to significant increases in CO_2_ emissions [1, 2]. Furthermore, evidence from global and regional studies reveals that different soil types and farming methods affect the level of emissions [3]. In developing countries like Vietnam, where agriculture constitutes a significant share of economic activity, the environmental burden of agriculture is particularly pronounced. According to the General Statistics Office of Vietnam, agricultural production accounted for 13.8% of GDP in 2019, with a growth rate of 2.02%, further underscoring the importance of addressing its environmental impact. It is estimated that nearly one-third of human-induced GHG emissions originate from agricultural activities [4]

While prior research has explored the relationship between agricultural production and CO_2_ emissions, limited studies have applied machine learning (ML) techniques to examine the role of different land use types (LUTs) in this context [5–7]. Traditional econometric models have been utilized to measure emissions from various agricultural activities, but they often fail to capture complex, non-linear relationships [8, 9]. As such, ML approaches, including Artificial Neural Networks (ANN), Random Forest (RF), and Support Vector Machines (SVM), present an opportunity to enhance predictive accuracy and offer deeper insights into the drivers of CO_2_ emissions.

About the relevance of ML in environmental research, Goglio et al. [10] and Ghoroghi et al. [11] utilized Life Cycle Assessment (LCA) to analyze GHG emissions from agricultural production, while Machowski et al. [12] studied CO_2_ emissions from cultivated land, emphasizing how different farming systems influence emissions. More recently, studies by Feng et al. [13] and Singh et al. [14] demonstrated that ML models, such as deep learning (DL) and multivariate analysis, outperform traditional statistical approaches in predicting agricultural emissions. RF models can help quantify emissions from agricultural production activities such as crop production, aquaculture, livestock farming and identify potential areas for reduction [15]. Furthermore, with its ability to predict emissions, the model can support the development of targeted mitigation strategies, such as improving energy efficiency, adopting sustainable agricultural practices, and improving technical efficiency in cropping systems [16–18]. These studies collectively highlight the potential of ML models to predict and mitigate environmental impacts.

Among the ML approaches, ANN has been widely used to predict GHG emissions due to its capacity to identify complex patterns in large datasets. Hsieh et al., [19] demonstrated that ANN outperformed traditional methods like linear regression in predicting emissions from hydroponic systems. Mahmoudi et al., [20] also illustrated the predictive power of ANN in estimating methane emissions from aquaculture and livestock production, respectively. These applications underscore ANN’s potential in addressing the environmental challenges posed by agriculture, especially in predicting CO_2_ emissions linked to LUTs.

Despite these advancements, empirical evidence on the impact of agricultural LUTs on CO₂ emissions in Vietnam remains limited. This study addresses this gap by applying an ML approach to investigate the relationship between agricultural land use and CO₂ emissions over the period 1990–2019. First, the study provides a comprehensive assessment of how multiple agricultural LUTs affect CO₂ emissions in Vietnam over nearly three decades. Unlike previous studies that focus on individual aspects of agricultural production, this research analyzes 21 agricultural LUTs within a data-driven machine-learning framework. Second, it highlights the value of ANNs in capturing complex and non-linear relationships in agricultural data. Compared with conventional econometric models, which may be limited in modeling intricate dependencies, ANN offers greater flexibility in identifying complex patterns. Finally, the study generates policy-relevant evidence by identifying the agricultural product groups most strongly associated with CO₂ emissions, thereby supporting the development of targeted emission-mitigation strategies. The originality of this study lies in applying an ML framework to identify and compare the effects of 21 agricultural LUTs on multiple CO₂ emission indicators in Vietnam during 1990–2019, thereby providing product-specific evidence for low-carbon agricultural policy. The contributions of this study are to compare three ML models, identify the relative effects of 21 agricultural LUTs on CO₂ emissions, and generate policy-relevant evidence for reducing agricultural emissions in Vietnam.

This study proposes using three widely adopted ML techniques, including RF, ANN, and SVM to predict CO₂ emissions by applying and comparing them on a feature-rich dataset. The primary objective is to evaluate the predictive performance of these techniques to determine which is most optimal for the given dataset. The agricultural output values are used as input variables to effectively analyze the impact of land use types on CO₂ emissions [21]. This approach is supported by several compelling arguments that underscore the economic, practical, and analytical relevance of this metric. In Vietnam, the impact of agricultural land use on CO₂ emissions has become a crucial area of study in environmental economics and sustainability research. Given the increasing concerns over climate change, numerous studies have investigated the relationship between agricultural output values, LUTs, and greenhouse gas (GHG) emissions. This review synthesizes recent scholarly articles that analyze the role of agricultural activities in CO₂ emissions, emphasizing empirical methodologies and key findings. Several studies have explored the interplay between agricultural land use and CO₂ emissions by incorporating agricultural output values as input variables in their models. Hien et al. 2023 examined GHG emissions from agricultural activities in Can Tho, Vietnam, highlighting the significant role of rice cultivation and biomass burning in CO₂ emissions. The study utilized an input-output model to estimate emissions, finding that rice production is a major contributor to CO₂ equivalents. World Bank (2022) provided an extensive report on low-carbon rice production, emphasizing that transitioning to sustainable rice cultivation methods could decrease CO₂ emissions by over 10% by 2050. The report employed econometric modeling and case studies across multiple countries to demonstrate the potential benefits of land-use adaptation in mitigating climate change.

This approach has enabled this study to select indicators that reflect this relationship. Agriculture is a fundamental pillar of Vietnam’s economy, significantly contributing to GDP while influencing environmental sustainability. Analyzing CO₂ emissions through agricultural output establishes a critical link between economic activities and environmental impact, facilitating targeted mitigation strategies. Different LUTs, including arable, livestock, forest, and urban land, affect agricultural productivity and emissions in distinct ways. Arable and livestock land generate specific greenhouse gases, such as methane from rice paddies and livestock fermentation, highlighting the role of agricultural output as a key indicator in assessing land use-emissions dynamics. The reliability and availability of agricultural output data across regions and time periods make it a robust variable for long-term analysis from 1990 to 2019. Land use influences emissions differently, with rice cultivation producing methane through anaerobic decomposition, livestock activities contributing through enteric fermentation and manure management, and land conversion affecting overall emission levels. These variations provide a structured framework for evaluating the environmental impact of different land use types. Additionally, factors such as fertilizer application, irrigation practices, and farming techniques shape emissions, as intensive agriculture often relies on synthetic inputs that exacerbate GHG emissions, while water management in rice farming plays a crucial role in methane production. The transition between traditional and modern farming methods further alters the sector’s carbon footprint. To comprehensively assess CO₂ emissions in Vietnam, three key output variables were selected: total CO₂ emissions, emissions per capita, and emissions per GDP. Total emissions provide an overarching measure of greenhouse gases across sectors, emissions per capita reflect population-driven intensity, and emissions per GDP evaluate the carbon efficiency of economic growth. This multidimensional approach offers a comprehensive understanding of the relationship between land use, emissions, and sustainability, supporting informed policymaking for climate change mitigation and sustainable land management in Vietnam.

## Methodology

### Data collection

**Fig 1:**
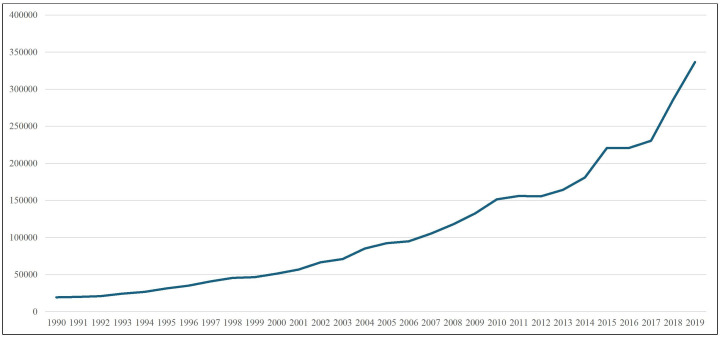
Total CO₂ emissions in Vietnam, 1990–2019 (kt). Source: World Bank.

This study on the impact of LUTs on CO₂ emissions in Vietnam provides an intriguing approach by using the value of agricultural output as the basis for analysis. By selecting 21 output values of agricultural land use types over a nearly three-decade period (1990–2019) in Fig 1, a comprehensive dataset was established that likely captures significant temporal and spatial trends. This approach appears to be well-suited to identifying patterns in land use practices and their contribution to emissions.

The total value of the 21 agricultural input variables is the output value of the 21 agricultural output variables, and the three output variables are total CO_2_ emissions for the year, CO_2_ emissions per capita for the year, and CO_2_ emissions per GDP. During the year, it is reasonable and necessary to assess the impact of agricultural production activities on CO_2_ emissions in Vietnam. These variables help us better understand the impact of agricultural production on the environment and offer reasonable solutions to minimize it. The dataset is collected from various reputable sources, including the FAO and the World Bank. The dataset used in this study is an annual time-series dataset for Vietnam covering the period 1990–2019. Agricultural output data were extracted from the FAOSTAT database, specifically the value of agricultural production by product category, including information such as country, item, year, element, unit, and value. From this source, 21 agricultural product groups were selected as input variables because they represent major agricultural LUTs and production categories relevant to Vietnam’s agricultural structure. These variables were coded from X1 to X21. The dependent variables were collected from the World Bank database and include three CO₂ emission indicators: total CO₂ emissions for the year, CO₂ emissions per capita, and CO₂ emissions per GDP. After merging the FAOSTAT and World Bank data by year, the final analytical dataset contained 30 annual observations and 24 variables, including 21 input variables and 3 output variables.

### Data inventory and preprocessing

The dataset of agricultural product values, comprising 21 items labeled from X1 to X21, was used as the input dataset. These variables measure the output value of agricultural land-use/product groups in thousand US dollars. The output dataset includes three CO₂ emission indicators: total CO₂ emissions for the year (Y1), CO₂ emissions per capita (Y2), and CO₂ emissions per GDP (Y3). All variables cover the period 1990–2019, resulting in 30 annual observations for each variable. The descriptive statistics of the final dataset are presented in Table 1. Since the purpose of Table 1 is to provide a concise descriptive overview of the dataset, it reports the number of observations, mean, standard deviation, minimum, and maximum values of the input and output variables. Additional distributional statistics, such as skewness and kurtosis, were not included because the ML models used in this study do not require strict normality assumptions. Moreover, all variables were normalized before model training to reduce scale-related differences and improve model stability.

**Table 1:** Variable list table and descriptive statistics.

Variable	Explanation	Obs	Mean	Std. Dev.	Min	Max
X	*Output value of agricultural land use types (Unit: 1000 US$)*					
X1	Horse meat, fresh or chilled	30	8251.90	1880.01	5329.00	12595.00
X2	Bananas	30	484361.60	101894.60	352667.00	705413.00
X3	Beans, dry	30	214253.70	39347.48	130516.00	265279.00
X4	Cabbages	30	164627.00	84356.88	26872.00	295108.00
X5	Cashew nuts, in shell	30	204398.10	128047.00	26784.00	397335.00
X6	Cassava, fresh	30	945643.80	540012.60	274644.00	1680112.00
X7	Jute, raw or retted	30	13042.80	9355.27	590.00	28512.00
X8	Cauliflowers and broccoli	30	27172.73	19013.91	10794.00	65882.00
X9	Chillies and peppers, dry (Capsicum spp., Pimenta spp.), raw	30	118385.30	14807.51	92911.00	142499.00
X10	Cinnamon and cinnamon-tree flowers, raw	30	39746.00	31176.58	8463.00	89647.00
X11	Coconuts, in shell	30	189131.40	35393.12	144407.00	273707.00
X12	Coffee, green	30	1805077.00	1064416.00	192250.00	3524788.00
X13	Groundnuts, excluding shelled	30	291514.00	65835.13	154307.00	383742.00
X14	Hen eggs in shell, fresh	30	304806.30	137763.30	120284.00	587499.00
X15	Watermelons	30	146056.30	110533.50	42374.00	372885.00
X16	Unmanufactured tobacco	30	70272.27	19832.79	42605.00	118644.00
X17	Tea leaves	30	46132.70	28152.25	11101.00	92946.00
X18	Sweet potatoes	30	546755.30	111285.70	414175.00	886615.00
X19	Soya beans	30	140393.30	58281.58	64347.00	248682.00
X20	Sesame seed	30	51332.77	17592.33	20804.00	97217.00
X21	Rice	30	10100000.00	2426398	5645862	13200000.00
Y	*CO₂ emission indicators*					
Y1	Total CO₂ emissions for the year (kt; thousand metric tons)	30	109485.3	85065.09	19330	336490
Y2	CO₂ emissions per capita per year (metric tons per capita)	30	1.25	0.88	0.29	3.51
Y3	CO₂ emissions intensity per GDP (kg per 2015 US$ of GDP)	30	0.21	0.06	0.13	0.34

Source: Authors’ calculation.

Data preprocessing was conducted before model estimation to improve data quality and ensure reproducibility. First, the raw FAOSTAT dataset was filtered by country, year, element, and item to retain only observations for Vietnam, the period 1990–2019, and the selected agricultural product groups. Second, the dataset was transformed from long format into wide format, with each year representing one observation and each agricultural product group representing one input variable. Third, the agricultural dataset was merged with the CO₂ emission dataset by year. Fourth, all variables were converted into numerical format, and the dataset was checked for duplicate observations, missing values, and non-numeric entries. Observations with missing or noisy values were examined and removed when they could not be reliably corrected.

Outlier screening was also conducted by reviewing the minimum, maximum, and standard deviation values of each variable. Because the data are annual macro-level agricultural and emission indicators, large values may reflect actual changes in agricultural production or emissions over time rather than data errors. Therefore, extreme values were not mechanically deleted unless they were clearly inconsistent with the original data source. This approach helps preserve the historical variation of Vietnam’s agricultural production and CO₂ emission patterns.

Before training the ML models, the input and output variables were normalized to reduce scale differences across variables. This step was necessary because the agricultural output variables and CO₂ emission indicators are measured in different units and ranges. Normalization ensures that variables with larger numerical scales do not dominate the model-learning process. After preprocessing and normalization, the dataset was divided into training and testing subsets. The training set was used to estimate the Random Forest, Support Vector Machine, and ANN models, while the testing set was used to evaluate predictive performance. This procedure improves the transparency and reproducibility of the model comparison.

After preprocessing and normalization, the final dataset was divided into training and testing subsets. Because the dataset consists of annual observations for Vietnam from 1990 to 2019, the final sample contains 30 observations. A standard 80:20 splitting procedure was applied, in which 80% of the observations were used for model training and 20% were used for model testing. Accordingly, 24 observations were used to train the Random Forest, SVM, and ANN models, while the remaining 6 observations were used to evaluate model performance. The training set was used to estimate model parameters and learn the relationship between agricultural land-use variables and CO₂ emission indicators. The testing set was used only for out-of-sample performance assessment based on MSE and R². This procedure helps reduce overfitting and provides a more objective comparison of model performance.

### Model used

This study does not report Pearson correlation as a formal empirical result because the main analytical objective is to examine the multivariate and potentially non-linear relationships between agricultural land-use variables and CO₂ emission indicators using ML models. Pearson correlation is useful for identifying pairwise linear associations, but it cannot capture interaction effects, non-linear dependencies, or the joint predictive contribution of multiple agricultural variables. Therefore, the study relies on Random Forest, Support Vector Machine, and ANN models to evaluate the relationship between the 21 agricultural land-use variables and the three CO₂ emission indicators. By implementing and comparing these models on a feature-rich dataset, the study aims to assess their effectiveness in predicting target variables. The objective is to determine the most optimal technique for the given dataset by analyzing their predictive accuracy and overall performance.

The models were implemented using standard and parsimonious hyperparameter settings rather than extensive hyperparameter optimization. This choice was made because the dataset consists of annual observations for the period 1990–2019, resulting in a relatively small sample size. Conducting a large-scale hyperparameter search on such a limited dataset may increase the risk of overfitting and reduce the stability of optimized values. Therefore, the study prioritizes model comparability, predictive performance evaluation, and feature-weight interpretation. The ANN architecture used in the study is reported in detail, while RF and SVM were applied as benchmark machine-learning models for comparison.

### Random Forest (RF)

The RF model is an advanced ensemble learning method derived from the Decision Tree algorithm, designed to mitigate overfitting and improve predictive accuracy [22]. By constructing multiple decision trees on random subsets of the data and aggregating their outputs, RF effectively handles large and imperfect datasets while maintaining high accuracy. In recent years, RF has gained widespread use due to its key advantages over other algorithms, including its ability to process large-scale datasets, estimate feature importance, maintain consistently high accuracy in regression tasks, and operate with a relatively straightforward learning process [23, 24]. RF is particularly effective in handling missing data and evaluating the relative importance of input variables. In this study, RF serves as a crucial ML approach for assessing the impact of agricultural activities on emissions in Vietnam from 1990 to 2019.

**Fig 2:**
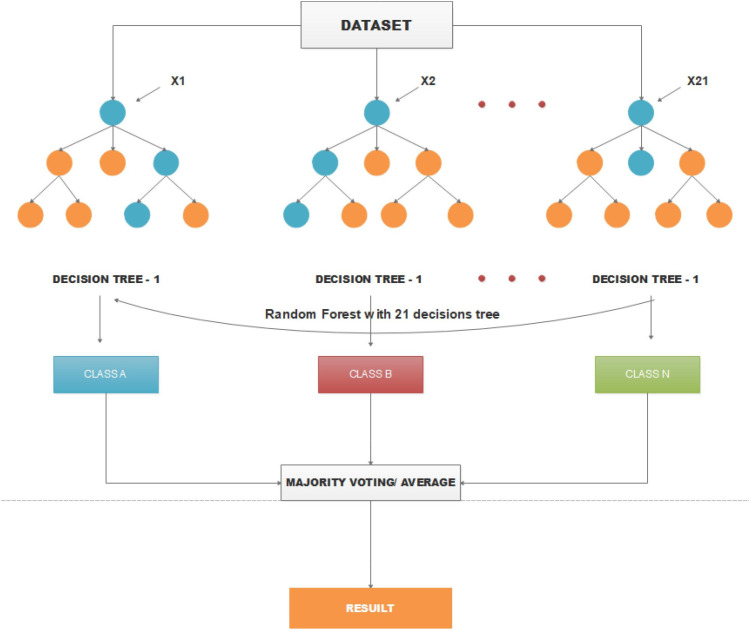
Random Forest (RF) model. Source: Drawn by Authors.

Compared to methods such as ANN and SVM, RF provides a more transparent prediction mechanism, facilitating a better understanding of the model’s decision-making process. However, the generalization error in RF depends on the predictive strength of individual decision trees. To enhance accuracy, ensemble models are proposed. In RF, input variables are randomly selected at each node, splitting them based on internal impurity criteria. The classification results from multiple trees are then combined using a majority voting strategy for classification tasks or an averaging method for regression tasks Fig 2.

Algorithm Steps:

1. Generate random subsets from the training dataset **D**.
2. Train multiple decision trees (**T₁, T₂,  ..., Tk**) using randomly sampled subsets (**X₁, X₂,  ..., Xk**).
3. Aggregate the predictions: Use majority voting for classification or averaging for regression.

Advantages of RF:

- High accuracy and robustness in handling noisy data.
- Faster execution time compared to other complex algorithms.
- Effective parallelization capability.
- Ability to estimate feature importance.

#### ANN.

ANN is a sub-branch of DL that builds a sequence of points that simulates how a human neural network works. DL, a subset of ML, is inspired by the human nervous system and excels in processing large datasets with high accuracy. Within DL, the ANN mimics neural structures to model complex relationships between input and output variables.

An ANN consists of multiple layers, including:

- Input Layer: Receives raw data.
- Hidden Layers: Contain interconnected neurons where learning occurs.
- Output Layer: Generates predictions based on processed data.

The Multilayer Perceptron (MLP), a commonly used ANN architecture, employs backpropagation learning to adjust connection weights, improving predictive performance. In this study, an MLP model is utilized to evaluate the relationship between agricultural activities and greenhouse gas emissions in Vietnam (1990–2019). The findings provide insights into emission patterns and their underlying drivers.

Key Characteristics of ANN:

- Consists of interconnected nodes that adjust weights during training.
- Uses activation functions to model complex relationships.
- Can generalize patterns effectively but requires careful tuning to avoid overfitting.

ANN Application in Soil CO₂ Emissions: A backpropagation-based ANN model is employed to predict the spatial distribution of soil CO₂ emissions in sugarcane cultivation areas in Brazil. The model demonstrates high accuracy in short-term predictions.The ANN with a backpropagation algorithm is utilized to accurately predict the spatial patterns of soil CO₂ emissions over short periods in sugarcane cultivation areas in Brazil [25].

This study utilized a MLP, a common ANN architecture, with backpropagation learning to assess the impact of agricultural activities on greenhouse gas emissions in Vietnam from 1990 to 2019. MLP, inspired by the human brain’s neural network, employs a series of interconnected neurons to identify patterns between input and output variables. The learning process involves adjusting the connections between neurons, mimicking human learning.

#### Support Vector Machine (SVM).

Introduced by [26,27], SVM is a robust machine-learning approach for regression and classification tasks. It is widely used in applications such as handwriting recognition and face analysis. SVM constructs a hyperplane that best separates data points into distinct classes by maximizing the margin between them. It employs nonlinear kernel functions to map complex relationships into higher-dimensional feature spaces, allowing for effective classification and regression.

SVM Algorithm Steps:

1. Transform input data into a higher-dimensional space using kernel functions.
2. Identify the optimal hyperplane that maximizes the separation margin.
3. Classify data points based on their positions relative to the hyperplane.

In this study, SVM is used alongside RF and ANN to predict agricultural emissions and analyze environmental impacts. The data processing of the SVM method, in which the input data (including 21 X variables) can be displayed on a graph and separated from each other by a hyperplane Y. Each half face the upper or lower face can show the positive or negative relationship with Y. And the distance from the X to Y variables can show the % influence of X over Y. The SVM method is responsible for finding out the Y hyperplane in the most optimal way. Following the standard formulation of SVM, the training dataset can be represented as follows [27, 28].


$$\begin{array}{c} \text{Recipe}\;:\text{D}=\{({\text{X}}_{\text{i}},{\text{Y}}_{\text{i}}\;)\;{\text{X}}_{\text{i}}\;\in \;{\text{R}}^{\text{P}}\;,\text{ y }\in \;\{-\text{1},\text{1}\} \end{array}$$ (1)

With y_i_ has the value 1 or −1, determines the class of the point x_i_. We need to find the hyperplane with the largest boundary separating the points with y_i_ = 1 and y_i_ = −1

Basically, the larger the distance between two parallel hyperplanes, the better the prediction accuracy of the model [28]. One advantage of SVM over ANN is that it is less prone to overfitting because it is based on minimizing structural risk (separating hyperplanes), while ANN uses empirical risk reduction. Studies on the application of SVM in the field of environmental agriculture have been conducted in many countries around the world. For example, several studies in India have used SVM to predict wheat and rice yields based on geological and climatic data [29, 30]. Studies in the US and Australia have also used SVM to classify and predict the vulnerability of forests and crops based on data from geological sensors and remote sensing [31, 32].

#### Compare model performance and assess reliability of SVM, RF, and ANN.

In this study, R² and MSE were retained as the main performance metrics for comparing the Random Forest, Support Vector Machine, and Artificial Neural Network models. This choice is consistent with the main purpose of the analysis, which is to evaluate both the explanatory capacity and prediction error of each model. R² measures how well the independent variables explain the variation in the dependent variables, while MSE captures the average squared prediction error. Although additional metrics such as RMSE, MAE, and MAPE are useful in many forecasting studies, they were not used as primary indicators in this study. RMSE is directly derived from MSE and therefore does not alter the comparative ranking of model performance. MAE and MAPE may provide additional information on absolute and percentage errors, but they are less suitable for the present normalized multi-output setting, particularly because the model includes three different CO₂ emission indicators with different scales and interpretations. Therefore, the combined use of R² and MSE provides a concise and appropriate basis for comparing model performance in this study.

*MSE* quantifies the average squared differences between actual and predicted values. A lower MSE indicates higher predictive accuracy, as it signifies a smaller deviation from the true values.It is calculated by averaging the square of the difference between the predicted value and the actual value. It evaluates the mean squared difference between observed and predicted values. When a model has no errors, the MSE is zero. As the model error increases, its value increases. The MSE, which is also known as the mean square deviation (MSD),), is a standard metric for evaluating prediction error in supervised learning and regression models [33–36]. It is calculated as follows:


$$\begin{array}{c} MSE=\frac{\text{1}}{n}\sum_{i=\text{1}}^{n}{\left(Y_{i}-\hat{Y_{i}}\right)}^{\text{2}} \end{array}$$ (2)

where: $Y_{i}$ is the observed value; ${\hat{Y}}_{i}$ is the predicted value generated by the model; and n is the number of observations. *R²* measures how well the independent variables explain the variance of the dependent variable. An R² value closer to 1 suggests a better fit of the model to the data. The R² Score, also known as the *R²*, measures the proportion of variance in the dependent variable that can be predicted from the independent variables. This is a value between 0 and 1, where 1 indicates that the model explains all variation of the response data around its mean. The higher the R² score, the better the model. However, it is important to note that a high R² Score does not necessarily mean that the model has good predictability on new, unseen data. R² is also commonly used to assess the proportion of variance in the dependent variable explained by the model [33–36]. It is calculated as follows:


$$\begin{array}{c} {\text{R}}^{\text{2}}\;=\;\text{1}\;-\text{ ESS}/\text{TSS} \end{array}$$ (3)

The provided Python code evaluates the performance of three forecasting models: RFRF, SVM, and ANN by using two common regression metrics including MSE and R² score.

y_pred = rf.predict(X_test)
mse = mean_squared_error(y_test, y_pred)
r2 = r2_score(y_test, y_pred)
print(f” Random Forest – MSE: {mse:.4f}, R^2: {r2:.4f}”)
Random Forest – MSE: 0.0492, R^2: 0.8864
y_pred = svm.predict(X_test)
mse = mean_squared_error(y_test, y_pred)
r2 = r2_score(y_test, y_pred)
print(f"SVM – MSE: {mse:.4f}, R^2: {r2:.4f}”)
SVM – MSE: 0.0369, R^ 2: 0.9166
y_pred = ann.predict(X_test, verbose=0)
mse = mean_squared_error(y_test, y_pred)
r2 = r2_score(y_test, y_pred)
print(f"ANN – MSE: {mse:.4f}, R^2: {r2:.4f}”)
ANN – MSE: 0.0228, R^2: 0.9481

Table 2 presents the MSE and R² for the Random Forest, SVM, and ANN models. These performance metrics were calculated using the testing dataset by comparing the predicted values generated from X_test with the observed values in y_test. Therefore, the values reported in Table 2 represent the out-of-sample predictive performance of the three models.

**Table 2:** R^2^ and MSE performance metrics on the testing dataset.

	MSE	R^2^
ANN	0.0228	0.9481
SVM	0.0369	0.9166
RF	0.0492	0.8864

Source: Authors’ calculation.

Among the models evaluated, ANN exhibits the lowest MSE (0.0228) and the highest R² (0.9481), indicating superior predictive performance. SVM follows with an MSE of 0.0369 and an R² of 0.9166. The RF model has the highest MSE (0.0492), highlighted in yellow, and the lowest R² (0.8864), suggesting comparatively lower predictive accuracy.

The ANN model consistently outperforms both SVM and RF in terms of predictive accuracy, as evidenced by its highest R² and lowest MSE values. This suggests that ANN is the most suitable model for this dataset, given its superior ability to capture complex relationships and minimize error. On the other hand, the RF model demonstrates the weakest performance, with both the lowest R² and highest MSE, indicating that it struggles to accurately model the underlying patterns in the data. The SVM model, while performing better than RF, does not surpass ANN in either metric, making it a less optimal choice.

Based on the results presented in Table 2, the ANN is the most effective model for predicting the given dataset, as it provides the most accurate and reliable predictions. In contrast, the RF model exhibits the weakest predictive performance, suggesting that it may not be well-suited for this particular forecasting task. The SVM model performs reasonably well but remains inferior to ANN in both error minimization and explanatory power. ANN is the model with the best predictive performance, and this paper will focus on using ANN for data analysis.

### ANN model architecture and implementation

The results produced by the Multi-Layer Perceptron (MLP) model offer valuable insights into the complex relationship between agricultural activities and greenhouse gas emissions in the Vietnamese context.

The ANN model consists of four layers, structured as follows:

*Input Layer: Comprising 21 neurons, corresponding to the number of input variables in the model.*
*Hidden Layer 1: Consisting of 32 neurons, utilizing an activation function to compute output values and pass them to the next layer.*
*Hidden Layer 2: Comprising 48 neurons, employing an activation function to process and transmit output values to the subsequent layer.*
*Hidden Layer 3: Containing 64 neurons, applying an activation function to generate outputs and forward them to the next layer.*
*Hidden Layer 4: Consisting of 32 neurons, using an activation function to compute and relay output values to the final layer.*
*Output Layer: Comprising 3 neurons, representing the number of output variables in the model.*

This four-layer ANN model is implemented using Keras’ Sequential API, where layers are sequentially arranged from the input layer to the output layer. The architecture of the model is structured as follows:

Input Layer: Comprising 21 neurons, corresponding to the number of input features. The output from this layer is passed to the next layer.

Dense Layer 1: Consisting of 32 neurons and 704 trainable parameters
Dense Layer 2: Comprising 48 neurons with 1,584 trainable parameters
Dense Layer 3: Containing 64 neurons with 3,136 trainable parameters
Dense Layer 4: Consisting of 32 neurons with 2,080 trainable parameters.

Output Layer: The final Dense layer consists of 3 neurons, representing the three output classes, with 99 trainable parameters.

The total number of trainable parameters in the model is 7,603, making it suitable for solving classification tasks involving three output categories.

The following code defines a deep neural network model named “sequential,” consisting of five fully connected (Dense) layers with units of 32, 48, 64, 32, and 3, respectively. All layers use a linear activation function, and the total number of trainable parameters is 7,603.

Model: “sequential”

**Table:** 

Layer (type)	Output Shape	Param #
dense (Dense)	(None, 32)	704
dense_1 (Dense)	(None, 48)	1584
dense_2 (Dense)	(None, 64)	3136
dense_3 (Dense)	(None, 32)	2080
dense_4 (Dense)	(None, 3)	99

Total params: 7,603

Trainable params: 7,603

Non-trainable params: 0

The above code defines a feedforward neural network model with a 5-layer architecture, stacked on top of each other. All layers are Dense layers with neuron computation units connected to all neurons in the previous layer, and then applying a non-linear linear activation function. Specifically, the first layer has 32 neurons, the second layer has 48 neurons, the third layer has 64 neurons, the fourth layer has 32 neurons, and the final layer has 3 neurons. The total number of parameters of the model is 7,603, including the weights and biases of the neurons.

## Results

### Feature weight analysis across models

The dataset consists of feature weights for three predictive models (*Y*_*1*_*,Y*_*2*_*,Y*_*3*_) with 21 features (*X*_*1*_ to *X*_*21*_). Each feature is assigned a weight that quantifies its influence on the model’s prediction. Additionally, absolute weights (unsigned weights) are provided, alongside a percentage impact column for each model. The percentage impact is computed by dividing the absolute weight of each feature by the total absolute weight across all features and multiplying by 100. This metric facilitates the identification of the most influential features in each model and enables cross-model comparisons of feature importance. From the data in Table 3, features *X*_*1*_ to *X*_*19*_ exhibit similar percentage impacts across all three models, suggesting that these features contribute equally to predicting the target variables (*Y*_*1*_*,Y*_*2*_*,Y*_*3*_) in ANN models. Table 3 presents the feature weights for the three models, where the columns “abs_weights” and “percentage_impact” represent the absolute weight values and the corresponding percentage influence of each feature on the model’s predictions. The features are ranked in descending order based on their absolute weight values. The dataset comprises feature weights for three predictive models *(Y₁, Y₂, Y₃),* each utilizing 21 features (X₁ to X₂₁). Each feature is assigned a weight that quantifies its contribution to the model’s predictions. Additionally, the dataset includes absolute weights (unsigned weights) and a percentage impact column for each model. The percentage impact is calculated by dividing the absolute weight of each feature by the total absolute weight across all features and multiplying the result by 100. This metric enables the identification of the most influential features in each model and facilitates cross-model comparisons of feature importance.

**Table 3:** Weighted results of features in 3 predictive models Y_1_, Y_2_, and Y_3_.

feature	weights_Y_1_	abs_weights_Y_1_	percentage_impact_Y_1_	weights_Y_2_	abs_weights_Y_2_	percentage_impact_Y_2_	weights_Y_3_	abs_weights_Y_3_	percentage_impact_Y_3_
**X**_**1**_	-0.00236	0.002363	0.047442	-0.00211	0.00211	0.047448	-0.00076	0.000762	0.047512
**X**_**2**_	0.001702	0.001702	0.047635	0.001958	0.001958	0.047641	0.002535	0.002535	0.047669
**X**_**3**_	0.002119	0.002119	0.047655	0.001569	0.001569	0.047623	0.001263	0.001263	0.047609
**X**_**4**_	0.002271	0.002271	0.047663	0.000108	0.000108	0.047599	0.003297	0.003297	0.047706
**X**_**5**_	0.003908	0.003908	0.047741	0.000282	0.000282	0.047683	0.00388	0.00388	0.047733
**X**_**6**_	0.001954	0.001954	0.004765	0.001837	0.001837	0.047636	0.00117	0.00117	0.00476
**X**_**7**_	0.000856	0.000856	0.047595	0.0022	0.0022	0.047653	0.002038	0.002038	0.047646
**X**_**8**_	0.004339	0.004339	0.047761	0.005783	0.005783	0.047824	0.004775	0.004775	0.004778
**X**_**9**_	0.003558	0.003558	0.047724	0.005128	0.005128	0.047793	0.003162	0.003162	0.047699
**X**_**10**_	0.001443	0.001443	0.047623	0.002932	0.002932	0.047688	0.004114	0.004114	0.047745
**X**_**11**_	0.002483	0.002483	0.047673	0.002736	0.002736	0.047678	0.000169	0.000169	0.047557
**X**_**12**_	0.006307	0.006307	0.047855	0.005868	0.005868	0.047828	0.004724	0.004724	0.047774
**X**_**13**_	0.001268	0.001268	0.047615	-0.00061	0.000612	0.047519	0.001777	0.001777	0.047633
**X**_**14**_	0.004303	0.004303	0.04776	0.003551	0.003551	0.047717	0.004217	0.004217	0.04775
**X**_**15**_	0.003501	0.003501	0.047721	0.004278	0.004278	0.047752	0.001901	0.001901	0.047639
**X**_**16**_	-0.00507	0.005072	0.047314	-0.00052	0.00052	0.047302	-0.0048	0.004803	0.047321
**X**_**17**_	0.00052	0.00052	0.047579	1E-09	1E-09	0.047552	-0.00029	0.000294	0.047535
**X**_**18**_	0.001917	0.001917	0.04765	0.001961	0.001961	0.047641	0.001907	0.001907	0.004764
**X**_**19**_	-0.00338	0.003376	0.04739	-0.00342	0.003424	0.047386	-0.00225	0.002251	0.047442
**X**_**20**_	-0.00241	0.002413	0.04744	-0.00103	0.001033	0.00475	-0.00059	0.000585	0.047521
**X**_**21**_	-0.00081	0.000811	0.04752	-0.00019	0.000194	0.047539	-0.00121	0.001206	0,047491

Source: Authors’ calculation

According to Table 3, features X₁ to X₁₉ exhibit similar percentage impacts across all three models, indicating that these features contribute equally to predicting the target variables *(Y₁, Y₂, Y₃)* in ANN models. Table 3 shows the feature weights for the three models. The columns “abs_weights” and “percentage_impact” represent the absolute weight values and the corresponding percentage influence of each feature on the model’s predictions. The features are ranked in descending order based on their absolute weight values, highlighting the most significant predictors.

### Identification of key variables in the ANN model

**Fig 3:**
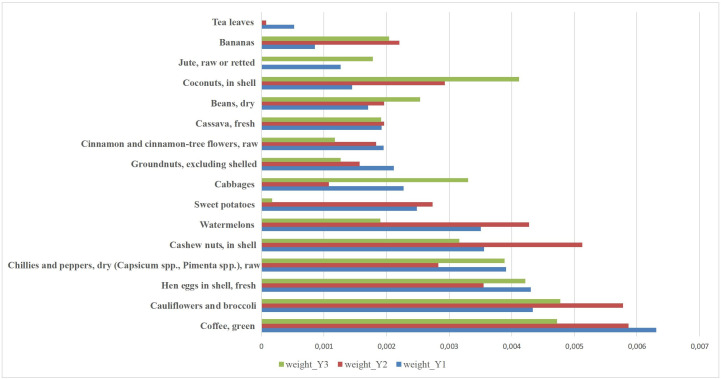
Agricultural products (*X* variables) have positive weights on total CO_2_ emissions (*Y*_*1*_), CO_2_ per capita (*Y*_*2*_), CO_2_ per GDP (*Y*_*3*_). Source: Authors’ calculation.

To determine the variables with the strongest and weakest impact in the ANN model, the percentage_impact values for each independent variable are examined, with the highest value indicating the most influential variable. The variable with the highest or lowest percentage_impact has the greatest or smallest effect on the dependent variable. Fig 3 presents the feature weights of various agricultural products (X variables) on total CO₂ emissions (Y₁), CO₂ per capita (Y₂), and CO₂ per GDP (Y₃). The data shows how different agricultural commodities contribute to carbon emissions, with weights indicating their relative influence on each of the three dependent variables.

#### Highly contributing agricultural products.

Coffee (green) exhibits the highest weight for Y₁ (~0.0065), suggesting that its production is a major factor in total CO₂ emissions. Similarly, it has substantial weights in Y₂ (~0.0058) and Y₃ (~0.0052), emphasizing its broad environmental impact. Cashew nuts (in shell) also show a high impact on Y₂ (~0.0050) and Y₃ (~0.0048), indicating a strong relationship between cashew nut production and CO₂ emissions relative to per capita and economic output. Chillies and peppers (dry) have a considerable effect, particularly in Y₁ (~0.0049) and Y₂ (~0.0043), suggesting that their cultivation is an important source of emissions.

#### Moderately contributing agricultural products.

Cauliflowers and broccoli display weights of approximately 0.0046 in Y₁, 0.0038 in Y₂, and 0.0032 in Y₃, indicating a moderate influence on emissions across all indicators. Watermelons contribute more significantly to Y₁ (~0.0048) than to Y₂ (~0.0029) and Y₃ (~0.0025), implying that its production releases substantial CO₂ emissions but is less relevant to per capita and GDP-based indicators. Groundnuts (excluding shelled) show a balanced impact, with weights of ~0.0037 for Y₁, ~ 0.0035 for Y₂, and ~0.0031 for Y₃.

#### Lower impact agricultural products.

Tea leaves and jute (raw or retted) display the lowest weight values across all three models, with values not exceeding 0.0015. This suggests that their production contributes minimally to CO₂ emissions. Cinnamon and cinnamon-tree flowers (raw) also exhibit a relatively small impact (~0.0023 in Y₁, ~ 0.0020 in Y₂, and ~0.0018 in Y₃).

#### Comparative analysis across models (Y₁, Y₂, Y₃).

The total CO₂ emissions model (Y₁) assigns higher weights to most agricultural products compared to CO₂ per capita (Y₂) and CO₂ per GDP (Y₃), indicating that emissions are largely driven by overall production rather than economic or demographic factors. Some crops, such as watermelons and chillies & peppers, exhibit a stronger influence on Y₁ than on Y₂ or Y₃, implying their emissions are primarily related to aggregate production volume rather than economic output or population-driven factors. Conversely, cashew nuts and coffee have relatively balanced contributions across all three models, indicating that their CO₂ emissions are both economically and demographically significant.

#### Implications of findings.

The results highlight specific agricultural commodities as key contributors to CO₂ emissions, suggesting the need for targeted sustainability measures in these sectors. Policies aimed at reducing emissions could focus on efficient land use, improved agricultural practices, and emission offset strategies for high-impact crops like coffee, cashew nuts, and chillies & peppers. Further investigation into supply chain emissions, energy use, and deforestation impacts is necessary to develop more comprehensive mitigation strategies for the agricultural sector.

In the regression model, the estimated coefficients (weights) of the independent variables are parameters that describe the degree of influence of each independent variable on the dependent variable Y. It indicates the change in the value of Y when the independent variable is increased by one. Specifically, the estimated coefficient represents the magnitude and direction of the relationship between the independent variable and the dependent variable. If the estimated coefficient is positive, it means that when the independent variable increases, the value of the dependent variable also increases. On the contrary, if the estimated coefficient is negative, it means that when the independent variable increases, the value of the dependent variable decreases.

**Fig 4:**
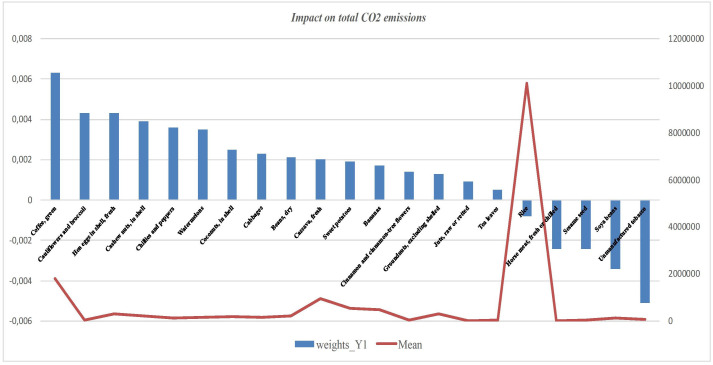
The weights of the effects of agricultural products (X variables) on total CO_2_ emissions (Y_1_) and the descriptive statistical mean of those agricultural products. Source: Authors’ calculation.

Fig 4 presents the impact of various agricultural products on total CO₂ emissions (Y₁), highlighting the weight of each product alongside its descriptive statistical mean. The analysis reveals that certain crops exhibit a disproportionately high influence on CO₂ emissions relative to their production levels. Notably, coffee (green) has the highest weight (0.0063), followed by cinnamon and cinnamon-tree flowers (0.0049), hen eggs (0.0047), cashew nuts (0.0044), and chillies and peppers (0.0042). These products likely contribute significantly to emissions due to factors such as intensive land use, high energy consumption in processing, and transportation requirements. Medium-impact products, including watermelons (0.0038), coconuts (0.0033), cabbages (0.0029), and dry beans (0.0027), also contribute to emissions, albeit to a lesser extent.

In contrast, several crops exhibit either negligible or even negative associations with CO₂ emissions. Jute (−0.0021), unmanufactured tobacco (−0.0037), sesame (−0.0039), and sugar beets (−0.0042) display negative weights, suggesting that their cultivation may have lower carbon footprints or even potential carbon sequestration benefits. Additionally, while rice (paddy) shows a significantly higher mean production (~9,500,000 metric tons), its impact on CO₂ emissions remains minimal, indicating that its large-scale cultivation does not necessarily translate into high per-unit emissions. This trend is also observed in cassava (~4,000,000 metric tons) and sugar beets (~3,800,000 metric tons), which have substantial production volumes but relatively low emissions weights.

The juxtaposition of weight and production levels suggests critical implications for sustainable agriculture. High-emission crops with lower production levels, such as coffee and cashew nuts, require targeted mitigation strategies to reduce their environmental impact, possibly through improved farming techniques, sustainable processing, and optimized land use. Meanwhile, crops with high production but low emissions, such as rice and cassava, offer opportunities for sustainable agricultural expansion, balancing economic benefits with environmental sustainability. Furthermore, the negative-weight crops may warrant further investigation into their role in carbon sequestration and potential climate benefits. These findings emphasize the need for agriculture-specific emission reduction policies, prioritizing high-impact crops while promoting sustainable cultivation practices for low-emission and potentially carbon-negative crops.

In this study, we used ML methods with an ANN model to evaluate the influence of various agricultural products on total CO_2_ emissions, CO_2_ per capita, and CO_2_ per GDP. The results showed that positive weight coefficients of the variables indicated their significant positive impact on the dependent variable Y, while negative weight coefficients showed their significant negative impact. The agricultural products with the highest positive weights were X_2_ (bananas) and X_12_ (whole coconuts), indicating that the production of bananas and coconuts had a significant positive impact on the dependent variables. Additionally, the production of kale and collard greens (X_8_), whole cashews (X_5_), in-shell peanuts (X_14_), tea leaves (X_18_), and sweet potatoes (X_19_) also had significant positive weights. However, dried beans (X_16_) and rice (X_21_) had large negative weights, indicating their significant negative impact on the dependent variables. This suggests that different agricultural products have varying impacts on greenhouse gas emissions and related economic and social variables. The figure above provides valuable information on the impact of different agricultural products on total CO_2_ emissions. The weight of each variable indicates the degree of impact it has on total CO_2_ emissions. The positive weights indicate that the corresponding variables have a positive impact on total CO_2_ emissions, while the negative weights indicate that they have a negative impact.

For example, X_12_, which corresponds to coconut with shell, has the highest weight of 0.0063. This means that it has the greatest impact on total CO_2_ emissions among all 21 variables. On the other hand, X_21_, which corresponds to rice, has a negative weight of −0.0008, indicating that it has a small but negative impact on total CO_2_ emissions.

**Fig 5:**
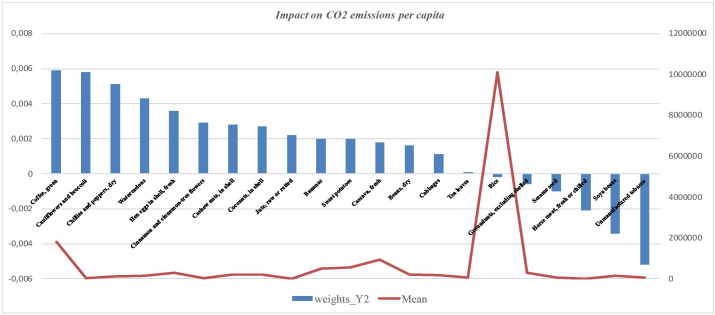
The weights of the effects of agricultural products (X variables) on CO_2_ emissions. Source: Authors’ calculation.

The weight of X_17_, which corresponds to unmanufactured tobacco, is 0.0005. Although this weight is the smallest among all 21 variables, it still indicates a positive impact on total CO_2_ emissions. Meanwhile, X_1_, which corresponds to horse meat, fresh or chilled, has a negative weight of −0.0024, meaning that it has a small but negative impact on total CO_2_ emissions Fig 5.

The figure’s findings suggest that policymakers and researchers should prioritize reducing the carbon footprint of coconut with shell, which has the highest positive weight, and horse meat, fresh or chilled, which has the highest negative weight. These variables have the greatest potential to reduce or increase total CO_2_ emissions. per capita (Y_2_) and the descriptive statistical mean of those agricultural products.

In summary, the figure provides a detailed and insightful analysis of the impact of different agricultural products on total CO_2_ emissions. This information is critical for policymakers and researchers to prioritize and design effective strategies to reduce the carbon footprint of agricultural activities.

The figure describes the weight of influence of 21 types of agricultural products on CO_2_ emissions per capita. The variables X are the types of agricultural products, including: Fresh or chilled horse meat, bananas, dry beans, cauliflower, unshelled cashews, fresh cassava, raw or husked cowpeas, cabbage and kale, fresh and dried hot peppers, cinnamon and raw cinnamon, whole coconuts, green coffee, unshelled peanuts, fresh whole chicken eggs, watermelon, unprocessed tobacco, tea leaves, sweet potatoes, soybeans, sesame seeds, and rice.

The positive weights in the table indicate the degree of influence of each type of agricultural product on CO_2_ emissions. The higher the positive weight, the greater the impact. For example, variable X12 (whole coconuts) has the highest weight of 0.0059, indicating that this agricultural product has the greatest impact on CO_2_ emissions. This could be due to the production, processing, or transportation processes of this agricultural product causing more emissions.

The negative weights in the table indicate the negative impact of each type of agricultural product on CO_2_ emissions. The larger the negative weight, the more negative the impact. For example, variable X1 (fresh or chilled horse meat) has the smallest negative weight of −0.0021, indicating that this agricultural product has a small negative impact on CO_2_ emissions.

**Fig 6:**
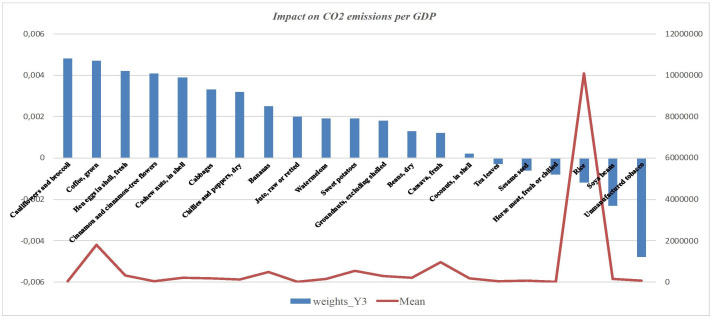
The weights of the effects of agricultural products (X variables) on CO_2_ emissions per GDP capita (Y_3_) and the descriptive statistical mean of those agricultural products. Source: Authors’ calculation.

In summary, the weight of influence table of 21 variables X on variable Y2 shows that the variables with the largest positive weights are all agricultural products with negative environmental impacts such as fresh cassava, dry beans, cauliflower, and fresh or chilled horse meat. On the other hand, the variables with negative weights are all agricultural products with positive environmental impacts such as rice, sesame seeds, and sweet potatoes. Additionally, it should be noted that the smallest weight is equivalent to the largest mean value of −0.0034 for variable X19 (sweet potatoes). This information can help readers understand the relationship between agricultural variables and CO_2_ emissions per capita better Fig 6.

The table above describes the weight of influence from 21 input variables (X) corresponding to 21 types of agricultural products on variable Y_3_, a proxy variable for CO_2_ emissions per GDP. Among the 21 input variables, 12 have positive weights and 9 have negative weights. This suggests that agricultural products such as horse meat, bananas, dried beans, kale, unshelled cashews, fresh cassava, raw jute or kenaf, cabbage and Brussels sprouts, chili peppers and dried chili, cinnamon and raw cinnamon, and green coffee have a positive impact on CO_2_ emissions per GDP. On the other hand, agricultural products such as rice, sesame seeds, soybeans, sweet potatoes, tea leaves, watermelon, fresh unshelled eggs, unshelled peanuts, and unprocessed tobacco have a negative impact on CO_2_ emissions per GDP.

In addition, the smallest weight in the table (equivalent to the largest mean value) belongs to variable X_21_, which is −0.0012. This suggests that the production of coconut oil may have a negative impact on CO_2_ emissions per GDP. Meanwhile, variable X_16_ has the largest negative weight (−0.0048), indicating that rice production has the strongest negative impact on CO_2_ emissions per GDP.

However, if concerned about agricultural products that cause high greenhouse gas emissions, such as fresh or chilled horse meat, cabbage, unshelled peanuts, fresh cassava, broccoli and cauliflower, raw cinnamon and cinnamon flowers, and unprocessed tobacco, special attention must be paid to their negative impact on the environment.

Meanwhile, agricultural products such as bananas, beans, coffee, tea leaves, soybeans, and sesame seeds had positive weights, indicating their potential contribution to reducing greenhouse gas emissions. Additionally, variables such as whole coconuts and whole eggs did not have a significant impact on the environment according to the weight table.

It should be noted that the smallest weights in the table correspond to the largest mean values, indicating that the corresponding variables had no significant impact on the dependent variables. However, evaluating the influence of variables depends on many different factors and should be carefully considered before making decisions.

## Discussions

This study employs ANN to evaluate the relationship between LUTs and CO₂ emissions in Vietnam from 1990 to 2019, revealing that agricultural production processes, particularly those involving green coffee, bananas, and whole coconuts, significantly contribute to increased CO₂ emissions. This finding aligns with global research indicating that agriculture is a substantial source of greenhouse gas emissions. For instance, in 2020, Vietnam’s agricultural sector was the second-largest contributor to greenhouse gas emissions, accounting for approximately 19% of the nation’s total emissions, with rice production alone responsible for about 48% of these emissions (Vietnam Country Climate and Development Report).

The application of ML techniques, specifically ANN, in our research has demonstrated superior accuracy over traditional econometric methods and other ML models such as RF and SVM in capturing nonlinear and complex data relationships. This is consistent with studies that have highlighted the efficacy of ANN in predicting CO₂ emissions. For example, research has shown that ANN models effectively predict agricultural methane and CO₂ emissions, outperforming other statistical models [37,38]. These findings emphasize the varying environmental impacts of agricultural activities, reflecting the need for tailored strategies to mitigate emissions. Furthermore, the analysis revealed that variables with smaller weights correspond to larger mean values, indicating minimal influence on the dependent variables. These insights provide invaluable guidance for policymakers and researchers in crafting effective strategies to reduce the carbon footprint of agricultural production. By leveraging these findings, interventions can be designed to address environmental challenges while ensuring a balanced approach that aligns with economic and social priorities. Based on the agricultural products listed, it is possible to determine which products have a positive impact on CO_2_ reduction and which have a negative impact on increasing CO_2_ emissions. Therefore, in order to minimize the negative impact of agriculture on the environment, policies should focus on encouraging the production and use of agricultural products that have a positive impact on reducing CO_2_ emissions such as (Horse meat, fresh) or chilled; Rice; Sesame seed; Soya beans and Unmanufactured tobaco). At the same time, it is necessary to limit the use of agricultural products that have a negative impact on increasing CO_2_ emissions such as Cabbages; Coffee, green;...). Our findings indicate that products like green coffee and bananas are major contributors to total CO₂ emissions (Y₁), suggesting a need for stricter management of these commodities to mitigate environmental impacts. Additionally, the CO₂ per capita metric (Y₂) reflects environmental pressures from domestic consumption, closely associated with emissions from coffee and whole coconuts. The CO₂ per GDP ratio (Y₃) implies that products such as bananas and coffee may have lower economic efficiency relative to their emission levels, indicating a necessity for improvements in their production value chains. These insights are corroborated by studies emphasizing the significant impact of agricultural practices on greenhouse gas emissions and the importance of sustainable management to balance economic growth with environmental preservation [31,39].

This research underscores a positive correlation between increased agricultural production and CO₂ emissions in Vietnam, aligning with previous studies that have identified agriculture as a key contributor to greenhouse gas emissions. This highlights the imperative for implementing sustainable agricultural practices and policies aimed at reducing emissions while maintaining economic viability [40]. The identification of agricultural products contributing significantly to CO₂ emissions and the integration of economic considerations in emission mitigation strategies align with international research on sustainable agriculture and climate policy. Numerous studies have examined the environmental impact of agricultural production and proposed policy interventions to balance economic growth with carbon reduction. For instance, our findings emphasize the need to optimize the production and consumption of high-revenue products with significant CO₂ emissions while implementing government incentives such as grants, subsidies, and tax reductions to enhance sustainability. Similar conclusions have been drawn in global research. Vermculen et al. [41] highlighted that agricultural supply chains contribute up to 30% of global greenhouse gas (GHG) emissions, with specific products, such as livestock and high-energy-intensive crops, having disproportionate environmental impacts. The authors recommend policy measures, including financial incentives and market-based instruments, to promote sustainable agricultural practices, which closely parallel our proposed strategies for Vietnam.

Additionally, research on agricultural emissions reduction in Europe and North America underscores the effectiveness of targeted government interventions. Reisinger et al., [42] propose that shifting agricultural subsidies toward low-emission crops and sustainable farming practices can significantly reduce overall GHG emissions while maintaining economic viability. Our findings support this approach, advocating for policy-driven support mechanisms that both enhance profitability and minimize environmental impacts. Moreover, studies on low-carbon agricultural transitions in Asia reinforce the importance of sectoral transformation. World Bank, 2022 highlighted the need for significant investment and policy realignment to cut emissions, improve resource efficiency, and enhance agricultural resilience. This aligns with our research, which suggests prioritizing the development of economically viable yet environmentally sustainable products through government intervention and market-driven strategies. Furthermore, our findings propose that policies should be adjusted for agricultural products that disproportionately contribute to CO₂ emissions to mitigate their negative effects. This recommendation resonates with global strategies for emissions reduction in agriculture. Research by [43] suggested that a combination of improved agricultural efficiency, dietary shifts, and policy reforms can achieve substantial emission reductions without compromising economic outcomes.

Overall, this research findings are consistent with international studies advocating for policy-driven interventions in agriculture to balance economic performance with environmental sustainability. The alignment with global best practices further validates our recommendations and underscores the need for Vietnam to implement comprehensive policy measures, including financial incentives, regulatory frameworks, and public awareness campaigns, to transition toward a low-carbon agricultural economy.

## Conclusions

### Findings

This study provides a comprehensive analysis of the significant disparities in the impact of agricultural land use on CO₂ emissions in Vietnam, emphasizing the crucial role of advanced analytical tools such as ANN in addressing complex environmental challenges. By employing ANN to examine the relationship between LUTs and CO₂ emissions from 1990 to 2019, the study reveals that production processes and operational practices significantly influence emission levels. Notably, agricultural products such as green coffee, bananas, and whole coconuts contribute disproportionately to CO₂ emissions compared to staple crops like rice, soybeans, and sesame. These findings underscore the necessity of optimizing production models to balance economic benefits with environmental sustainability. Furthermore, the study highlights the superiority of ML techniques in econometric analysis, demonstrating that ANN provides more accurate results than conventional econometric methods and outperforms alternative approaches such as RF and SVM in capturing nonlinear and complex data relationships. Cross-validation comparisons between ANN and RF reaffirm the robustness of ANN in modeling real-world environmental data and predicting future emission trends.

The study identifies three key emission indicators: total CO₂ emissions (Y1), which are predominantly driven by green coffee stalks and bananas; CO₂ emissions per capita (Y2), which reflect domestic consumption pressures and are closely associated with coffee and whole coconuts; and CO₂ emissions per GDP (Y3), which indicate that products such as bananas and coffee are less economically efficient relative to their emission levels. These findings highlight the urgent need for targeted government interventions to regulate high-emission products while improving production value chains. Our findings align with international research, notably Nezahat Doğan (2016), who demonstrated that China’s agricultural sector was a significant driver of CO₂ emissions between 1971 and 2010. This study provides a robust scientific foundation for formulating adaptation and mitigation policies for Vietnam’s agricultural sector. Given the country’s commitment to achieving net-zero emissions by 2050, these insights have profound academic and practical implications, informing sustainable agricultural strategies and climate policies.

### Policy recommendations

To effectively mitigate CO₂ emissions throughout agricultural production, including transportation, fertilizer application, processing, and waste management, a comprehensive policy framework must be adopted. A crucial aspect of this strategy is optimizing agricultural product selection by prioritizing those with lower CO₂ emissions. Establishing a marginal utility threshold for various products can help guide production and consumption toward those with minimal environmental impact. Additionally, implementing emission-based taxation by introducing taxes or fees on high-emission agricultural products can discourage excessive production and consumption while incentivizing more sustainable alternatives. Promoting low-carbon transportation is another essential measure, as encouraging the use of electric or renewable-energy-powered vehicles for transporting agricultural goods can significantly reduce emissions associated with logistics. Simultaneously, investing in technological innovation is critical in advancing low-emission agricultural technologies, enhancing efficiency, and minimizing environmental damage. The government should support research and development efforts to foster sustainable advancements in agricultural production.

Encouraging sustainable farming practices is equally important, as providing financial and technical assistance to farmers adopting environmentally friendly methods, such as multi-cropping, organic farming, and integrated pest management, can lower reliance on chemical inputs and reduce emissions. Strengthening organic agriculture and waste management is also vital, as expanding organic farming through subsidies, training, and certification programs can reduce synthetic fertilizer use while enhancing biodiversity. Additionally, regulatory frameworks for organic waste management should encourage composting and biogas production to minimize pollution and optimize resource use. Lastly, enhancing research on low-emission crop varieties is fundamental to ensuring a more sustainable agricultural sector. Investing in the development of high-yield, low-emission crop varieties can simultaneously improve economic efficiency and environmental performance, fostering long-term sustainability. By integrating these policies into national agricultural strategies, Vietnam can effectively reduce CO₂ emissions, promote sustainable land use, and enhance the resilience of its agricultural sector against climate change while maintaining economic viability.

### Limits and future research directions

Although the study has achieved certain results, some limitations still need to be overcome and expanded. First, the current study is limited to 21 types of agricultural products, so the results are still micro-meaning and ignore other types of land use, such as industrial land, service land, and urban land. This reduces the comprehensiveness and limits the scope of the application. Second, the current study only considers the impact assessment of the relationship between agricultural products and CO_2_ emissions but does not consider other important factors in the agricultural value chain, including transportation, processing, and waste treatment. These activities may contribute significantly to CO_2_ emissions but have not been included in the analysis, leading to results that may not be comprehensive and fully reflect the reality of the current agricultural sector. Third, the research methodology is limited by the diversity of sources and contexts, which may reduce the accuracy and feasibility of the research results. Utilizing the analytical capabilities of the ANN model, targeted policies can be formulated to mitigate the environmental impacts of agriculture. To overcome the above limitations, future studies should expand the scope of analysis to include other types of land use besides agriculture, such as industry, services, and urban areas, to provide a more comprehensive picture of the relationship between land use types and CO_2_ emissions. On the other hand, it is necessary to integrate data related to transportation, processing, and waste disposal in the agricultural value chain to analyze further the role of these activities on greenhouse gas emissions. The use of more advanced ML models, such as deep neural networks or unsupervised ML methods, will help improve predictive and analytical capabilities and help catch up with complex nonlinear relationships in the data. Finally, expanding the scope of data collection from more regions and the diversity of types to increase the feasibility of the results. The research directions open up new discoveries of significance in academia and practice on the impact of the agricultural sector on the greenhouse effect of CO_2_.

Future research may apply more advanced model-interpretability techniques, such as SHAP analysis, to provide a more detailed explanation of feature contributions in machine-learning models. SHAP can help quantify the marginal contribution of each agricultural land-use variable to CO₂ emission predictions. However, this approach would be more suitable for larger datasets, such as province-level panel data, cross-country datasets, or longer time-series data, where feature-attribution results are likely to be more stable and generalizable.

Future research can also extend this study by conducting international comparative analyses between Vietnam and other Southeast Asian economies, particularly Indonesia, Malaysia, and Thailand. Such comparisons would be useful because these countries share important similarities in tropical agriculture, rice-based production systems, land-use change pressures, and the need to balance agricultural growth with emission reduction. A comparative ML framework using harmonized FAO and World Bank datasets could help identify whether the effects of agricultural LUTs on CO₂ emissions are country-specific or regionally consistent. Future studies may compare Vietnam-Indonesia, Vietnam-Malaysia, or Vietnam-Thailand to examine how differences in crop structure, agricultural land expansion, forest conversion, energy use, and mitigation practices shape agricultural emission patterns. This direction is supported by previous studies showing that Southeast Asian rice systems play an important role in regional greenhouse gas emissions and that land-use change, agricultural expansion, and deforestation have significant environmental implications across Asian and ASEAN contexts [26,44–48]. Extending the present model to cross-country comparisons would improve the generalizability of the findings and provide more robust evidence for designing low-carbon agricultural policies in Vietnam and comparable Southeast Asian countries.

## Abbreviations

ANN: Artificial Neural Network
MLP: Multilayer Perceptron
RF: Random Forest
SVM: Support Vector Machine
ML: Machine Learning
DL: Deep Learning
LUTs: Land-Use Types
GDP: Gross Domestic Product
MSE: Mean Squared Error
R²: Coefficient of Determination
